# Surface and mantle records reveal an ancient slab tear beneath Gondwana

**DOI:** 10.1038/s41598-019-56335-9

**Published:** 2019-12-24

**Authors:** Guido M. Gianni, César Navarrete, Silvana Spagnotto

**Affiliations:** 1 Instituto de Estudios Andinos Don Pablo Groeber, UBA-CONICET, Departamento de Ciencias Geológicas, FCEN, Laboratorio de Geodinámica. Universidad de Buenos Aires, Buenos Aires, Argentina; 2 Instituto Geofísico Sismológico Ingeniero Volponi, Universidad Nacional de San Juan, San Juan, Argentina; 3 Laboratorio Patagónico de Petro-Tectónica, Universidad Nacional de la Patagonia “San Juan Bosco”, Dpto. de Geología, F.C.N, Chubut, Argentina; 4 FCFMyN, Universidad Nacional de San Luis–CONICET, San Luis, Argentina

**Keywords:** Geodynamics, Tectonics

## Abstract

Vertical slab-tearing has been widely reported in modern convergent settings profoundly influencing subduction and mantle dynamics. However, evaluating a similar impact in ancient convergent settings, where oceanic plates have been subducted and the geological record is limited, remains challenging. In this study, we correlate the lower mantle structure, which retained the past subduction configuration, with the upper-plate geological record to show a deep slab rupture interpreted as a large-scale tearing event in the early Mesozoic beneath southwestern Gondwana. For this purpose, we integrated geochronological and geological datasets with P-wave global seismic tomography and plate-kinematic reconstructions. The development of a Late Triassic-Early Jurassic slab-tearing episode supports (i) a slab gap at lower mantle depths, (ii) a contrasting spatiotemporal magmatic evolution, (iii) a lull in arc activity, and (iv) intraplate extension and magmatism in the Neuquén and Colorado basins. This finding not only has implications for identifying past examples of a fundamental process that shapes subduction zones, but also illustrates an additional mechanism to trigger slab-tearing in which plate rupture is caused by opposite rotation of slab segments.

## Introduction

Tear in subducting plates, linked to vertical or horizontal slab ruptures, are outstanding tectonic features described in some modern subduction^1,2^ and collisional^3^ settings. While horizontal oceanic slab-tearing is expected after continental collision^4^, vertical slab tearing is detected in convergent settings that undergo non-uniform slab retreat^2^ or in zones at the edges of active margins accommodating trenchward slab motion^1^. In addition, vertical tearing is locally observed where aseismic ridges^5^, mantle plumes^6,7^ or oceanic fracture zones interact with active margins^8^.

In contrast to horizontal tearing leading to slab break-off, often invoked to explain ancient post-collisional tectonomagmatic events^9^, the fingerprints of vertical slab tears are more difficult to detect. Identification of this process demands joint evidence from independent methodologies, such as geophysics, geochemistry and structural geology (e.g.^10,11^). This task is more difficult in ancient convergent settings where oceanic slabs have been fully subducted, and the geological record is cryptic and severely overprinted by shear zones. As a consequence, the tectonomagmatic characteristics of this process have only been accurately described in active margins in current subduction zones (e.g., Mediterranean region^10,12^; Aegean and Anatolia regions^13,14^; Tonga subduction zone^15^; Western South America^11,16^; Kamchatka subduction zone^17^; Marianas subduction zone^18^) and relatively young convergent margins (no older than the early Cenozoic^2,13,16,19^). Geophysical and geological observations and numerical and analog modeling studies have shown that slab-tearing directly influence along-strike subduction morphology and related mantle flow patterns^1,2,10^. Geological consequences of this process include acceleration of trench retreat^13,20^, creation of slab gaps and consequent mantle flow^21^, anomalous arc-backarc magmatism^2,10^, thermal perturbations^22^, and upper-plate segmentation and extension^13^. Slab gaps created by slab tears differ from those produced by subduction of spreading centers, which result from continued sublithospheric divergence of oceanic plate boundaries (i.e. not slab rupture) leaving a geological fingerprint widely detected in ancient settings. This process is commonly identified by its unique tectonomagmatic record associated with anomalous magmatism and hydrothermal activity in the forearc, a transient arc shut-off in the orogenic sector and extensive alkalic and/or tholeiitic OIB plateau basalts in the backarc area^23^.

**Figure 1:**
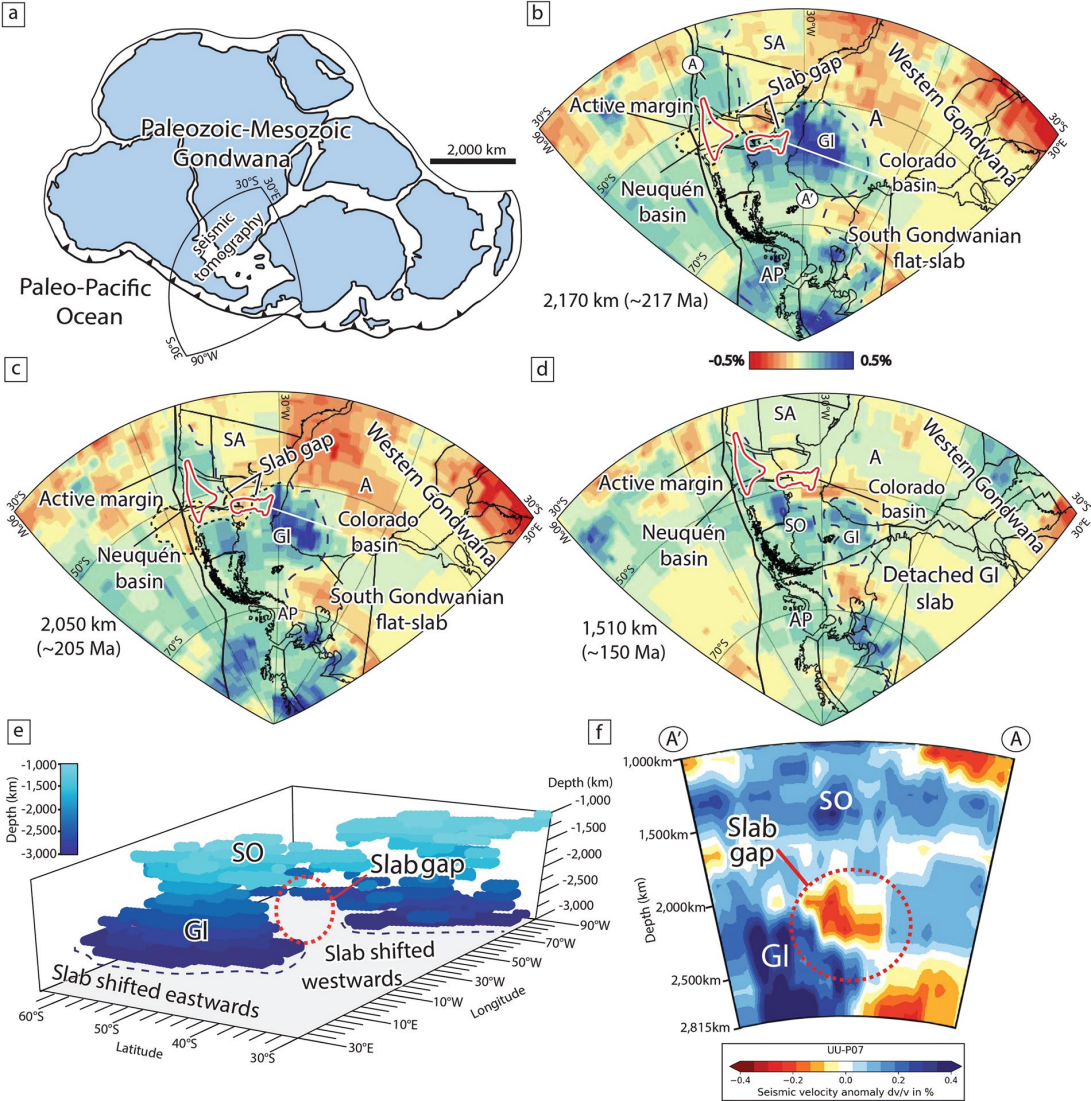
Reconstruction of the southwestern Gondwana margin during the Late Triassic to Jurassic and the lower mantle structure from the UU-P07 P-wave global seismic tomography model. (**a**) Paleogeographic reconstruction of Gondwana (Modified from Navarrete *et al*.^30^). Mantle tomography slices^33^, corresponding to the subducted slab at (**b**) ~217 Ma, (**c**) ~205 Ma and, (**d**) ~150 Ma with a lower mantle slab sinking rate of ~1 cm/yr^37^. Overlaid are plate reconstructions^31^ at (**b**) ~217 Ma, (**c**) ~205 Ma and, (**d**) ~150 Ma, respectively. (**e**) 3-D visualization of positive anomalies above >0.15% interpreted as fossil slabs^24,30,37^. (**f**) Cross section from the UU-P07 showing the slab gap inferred in this study. See profile location on (**b**). Abbreviations are A: Africa, AP: Antarctic Peninsula, SA: South America, GI: Georgia Island slab and SO: South Orkney slab.

The discovery that the lower mantle retains information about subduction configurations back to Mesozoic times^24–26^, opens new possibilities for understanding complex geodynamic processes at ancient convergent settings. We build on this key finding to track a major slab tear event beneath Gondwana during the Mesozoic (Fig. 1a). We integrate geochronological and geological datasets from continental areas corresponding to the southwestern Gondwana margin with P-wave global seismic tomography and Mesozoic plate-kinematic reconstructions. These combined data, support our hypothesis that a previous large-scale slab tear event can explain several seemingly unconnected geological and geophysical observations. The existence of an ancient slab rupture event clarifies the origin of an enigmatic upper-plate tectonomagmatic segmentation^27^ and a significant high-seismic velocity gap in the subducted slab. Moreover, our model sheds light on the origin of Late Triassic intraplate extension and magmatism associated with the opening of the Neuquén basin^28^, the most prolific oil basin of southern South America and the offshore Colorado basin^29^. More importantly, this exemplifies an additional mechanism that can trigger slab-tearing associated with the reconfiguration of the subduction system linked to changes in the subduction angle.

## Results and Discussion

### Slab-tearing from deep mantle to the surface record

**Figure 2:**
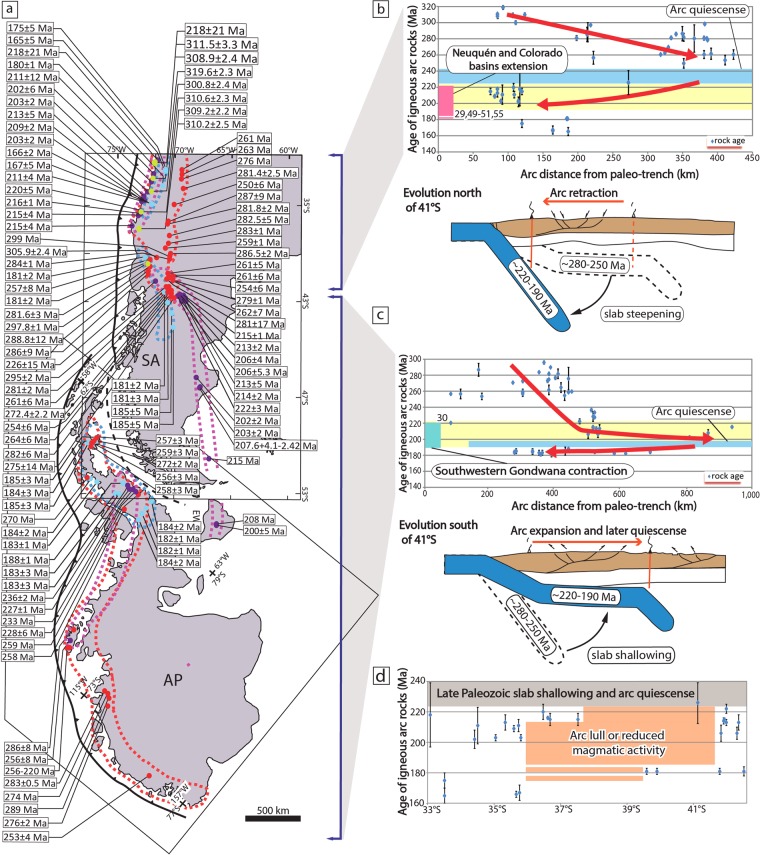
Spatiotemporal evolution of the Late Paleozoic-Mesozoic arc magmatic activity. (**a**) Compilation of geochronological data from subduction-related igneous rocks^30,40,41^. Dashed red, purple, and blue lines are the Late Carboniferous-Permian, Triassic, and Jurassic arc domains, respectively. (**b**,**c**) Are spatio-temporal analyses of the magmatic arc activity north and south of 41°S, respectively. Yellow bar indicates a reconfiguration of the subduction system starting at ~225–220 Myr. This event was associated with an expansion of magmatic activity and subsequent arc gap linked to the onset of flat-subduction south of 41°S^30^ and a simultaneous arc retraction to the north related to slab steepening(Refs. ^40–42^, this study). (**d**) Arc rocks age vs. latitude diagram showing the existence of a local lull or reduction in magmatic activity between 36°30′S and 41°S that took place from ~220 to 180–175 Myr related to the formation of a slab gap. Abbreviations are the same as in Fig. 1.

**Figure 3:**
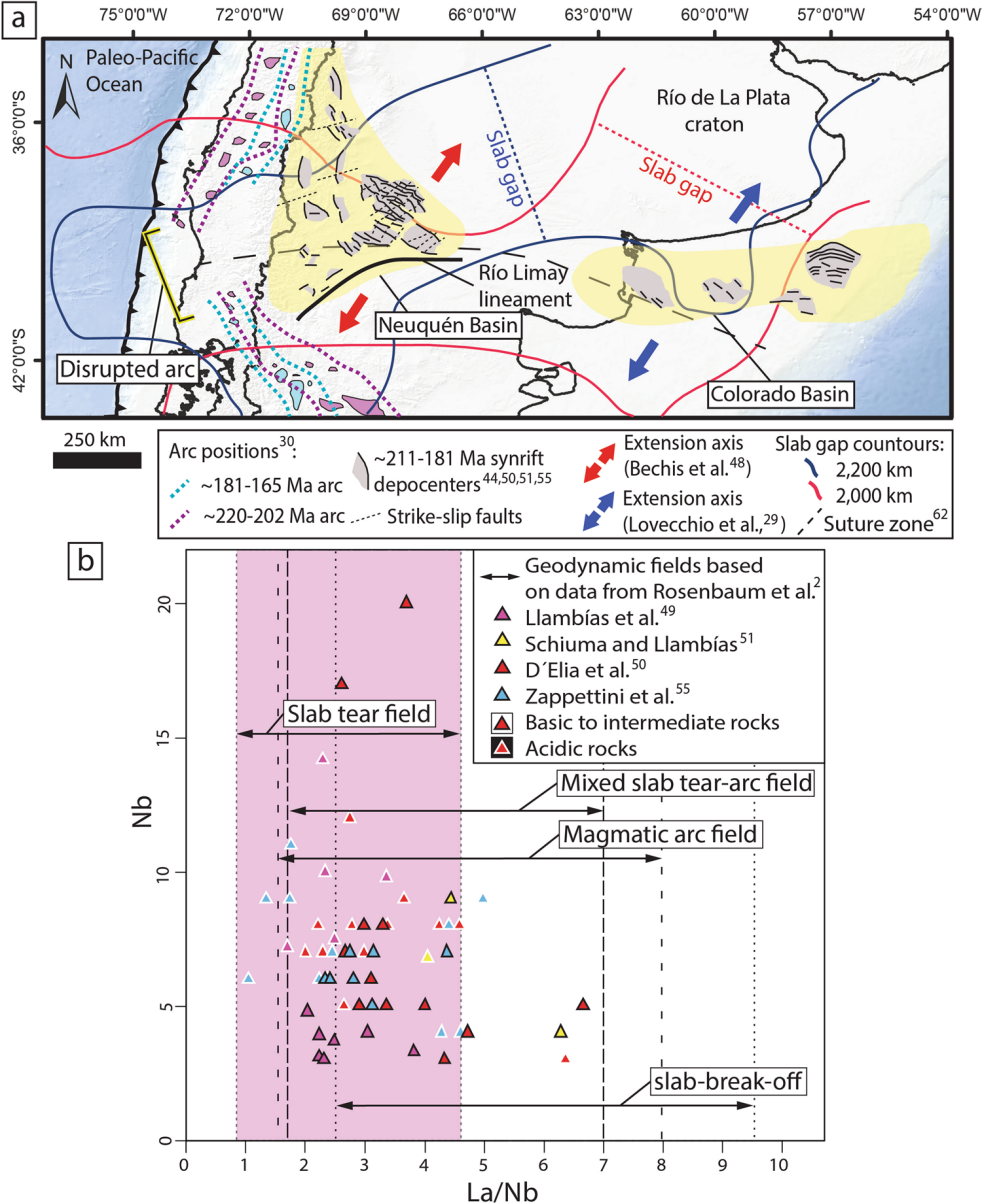
Tectonic setting of the southwestern Gondwana margin in the Late Triassic to Early Jurassic and Nb vs. La/Nb diagram to determine the tectonomagmatic setting of the Neuquén basin. (**a**) Late Triassic-Early Jurassic schematic tectonic map showing the area of arc lull reflected in a discontinuity in the magmatic arc belt, intraplate extension in the Neuquén and Colorado basins and the inferred area of the slab gap. (**b**) Nb vs. La/Nb diagram showing slab tear magmatism^2^ plotted with a compilation of geochemical data from the Pre-Cuyano synextensional unit^49–51,55^ that displays the potential influence of the formation of the slab gap in the magmatic activity of the Neuquén basin.

We start by analyzing the lower mantle structure to a depth corresponding to the subduction configuration in Late Triassic to Late Jurassic times^24,25^ below the southwestern Gondwana margin. We drew on the work of Navarrete *et al*. for our reconstruction, but focused on a previously overlooked slab gap. This approach is based on overlapping the reconstructed positions of southwestern Gondwana in Mesozoic times^31^ by using the GPlates 2.0 software^32^ with tomographic slices at lower mantle depths from the UU-P07 P-wave global seismic tomography model^33^. The lower mantle positive velocity anomalies in the tomographic model have been interpreted as fossil oceanic slabs and are thought to preserve former subduction positions^24,25^. Furthermore, the kinematic reconstruction^31^ helps to correlate the mantle structure with the geological record in the continental upper-plate. The UU-P07 P-wave seismic model has been previously used to build plate reconstructions in the Triassic^24,25^ and is generally chosen for analyses like ours^34^. As geological significance should only be attributable to a reliable mantle structure resolvable in multiple seismic tomography studies^35^, we create lower mantle vote maps^36^ with 24 global P-wave and S-wave tomography models that confirm the robustness and distribution of positive seismic anomalies, interpreted as fossil slabs, discussed below (Fig. S1 in supplementary material). We conduct an analysis of tomographic slices at 2170, 2050 and 1510 km. Assuming a whole-mantle slab sinking rate of ~1 cm/yr determined from the anomalies analyzed in this study^37^, these depths would correspond to subduction configurations at ~217 Ma, 205 Ma, and ~150 Ma, respectively (Fig. 1a–d). A slab sinking rate of ~1,2 cm/yr, within the range of previous estimates obtained from the worldwide tomographic analysis of subduction zones^26,36–39^, still yields a scenario roughly compatible with the geological record (Fig. S2 in supplementary material). A sinking rate greater than ~1,2 cm/yr does not correlate with the tectonic evolution discussed below. The general features in these reconstructions have been already identified by van der Meer *et al*.^24,25^ and further discussed by Navarrete *et al*. One of these mantle features is an elongated fast seismic anomaly coinciding with the reconstructed western Gondwana active margin^40^ located north of 50°S^30^ (Fig. 1b,c). This anomaly has been interpreted as a fossil slab subducted at a steep angle beneath southwestern Gondwana^30,40^ as implied by the record of contemporaneous accretionary wedge formation and arc magmatism in the Late Triassic to Early Jurassic^41,42^. The other mantle structure, is indicated by a fast anomaly referred to as the Georgia Island slab^37^ extending up to 2600–2800 km to the east of the reconstructed position of the Gondwana margin below Patagonia and the Antarctic Peninsula^25^ (Fig. 1b,c). This anomaly has also been interpreted as a fossil slab^25^ but associated with a large-scale flat-subduction configuration that explains the significant misfit with respect to the slab position in the north between ~220 and 190 Myr^30^ (Fig. 1b,c). The Georgia Island slab detached and sank sub-vertically sometime between 200 and 180 Myr below western Gondwana preserving its fossil position far from the plate margin^30,37^ (Fig. 1d). The contrasting subduction configuration along the active margin of Gondwana is independently supported by a spatiotemporal analysis of arc-related rocks, which is expected to correlate with slab dip^43^. For this analysis, we integrated three previous geochronological datasets^30,40,41^ (See methods section for further details) along the southwestern active margin of Gondwana (Fig. 2a). The spatiotemporal plot shows that north of 41°S arc activity between ~220 and 200 Myr concentrated near the paleo-trench^41,42^, while to the south coeval magmatic activity migrated eastward and eventually shut–off from ~200 to 190 Myr^30^ as expected in flat-subduction settings (Fig. 2b,c). Notably, we observe that these two anomalies are separated by a major fast velocity discontinuity (Figs. 1b–d,f and S1 of supplementary material). We interpret this mantle structure as a first-order segmentation in the fossil slab associated with the vestiges of a sub-vertical slab gap allowing for the existence of slab segments with contrasting dip angles. A continuous anomaly below the reconstructed margin can only be delineated in the mantle at shallower depths around 1500–1800 km, corresponding to the subduction of the mid-Jurassic South Orkney slab^30,37^ (Fig. 1d). Independent evidence of a Late Triassic-Early Jurassic slab gap is derived from along-strike analysis of the Mesozoic magmatic arc (Fig. 2d). Subduction segmentation in the Late Triassic is evidenced by a local reduction or lull in arc activity^27^ between 37°S and 41°S that took place from ~220 to 175 Myr (Figs. 2d and 3a). This process would have terminated between ~175 and 160 Myr as inferred from increased arc activity^27,28,44^, the appearance of the South Orkney Island slab in the Middle to Late Jurassic^30,37^, and the record of a sudden increase in back-arc subsidence interpreted as a dynamic effect triggered by subduction reactivation^45^ (Fig. 1d). The original slab gap morphology may depart from the original geometry due to spherical shell strain, a consequence of decreasing Earth volume with depth and thermal erosion in torn edges^46^. However, the close link to the geological record indicates that this feature has retained its original position since Mesozoic times.

The potential development of a ~220–175 Myr slab rupture explains additional aspects of the Mesozoic geological record of the southwestern Gondwana margin. Early to Middle Triassic extension took place roughly parallel to the active Gondwana margin, which is well preserved in the Northern and Central Andes^47^. In the Late Triassic, extension mostly ceased and basins entered a *sag* stage. However, to the south between 35°S and 41°S a roughly east-west intraplate extensional-transtentional system developed in relation to the opening of the Neuquén^27,48^ and Colorado basins^29^ (Fig. 3a). Intraplate deformation nucleated in a structural framework linked to late Paleozoic structures that was reactivated under a NE-SW tensional stress regime^29,48^ (Fig. 3a). The distributed character of intraplate extension in this region is interpreted as the result of a wide rifting mechanism controlled by pre-existing weak orogenic lithosphere^48–50^. Available surface geological information of this extensional stage mostly comes from the Neuquén basin^44,45,48–52^, while in the offshore Colorado basin this stage is only recognized from 2-D seismic reflection data^29^. The synextensional stage in the Neuquén basin is known as the Pre-Cuyano Cycle and is constrained between ~220 and ~181 Myr^44,51,52^. The extension in the Neuquén basin was accompanied by the shallow intrusion of igneous bodies, lava flows and pyroclastic deposits comprising up to 70% of the total synextensional infill^50^. In addition, this stage was associated with a Late Triassic-Early Jurassic thermal event that produced diastathermal metamorphism in synextensional units^53^. The origin of the Late Triassic-Early Jurassic extension in the Neuquén basin remains highly debated; while the origin of the Colorado basin is unknown^29^. In the former, proposed tectonomagmatic models include; passive extension due to gravitational collapse of a previously thickened crust^54^, active intraplate extension due to upwelling in the mantle of thermal^49^ or compositional origin^50^ or a combination of both mechanisms^27,50^. The intraplate extension was aided by slow or arrested subduction to the west and/or a coetaneous raise in intraplate stresses linked to the break-up of the Gondwana supercontinent^27,50^.

Geochemical data from volcanic rocks interfingered within the Late Triassic-Early Jurassic synextensional units in the Neuquén basin indicate a calc-alkaline character, and to a lesser extent tholeiitic with arc signatures in the base that evolve to intraplate signatures to the top^49,55^. According to Zapettini *et al*., the initial magmatic arc signature represents a metasomatic inheritance of the mantle produced by previous subduction stages. Trace element geochemistry in rocks belonging to the Pre-Cuyano synrift deposits indicate a mantle-derived origin^49,50^ with high crustal input^55,56^. Strikingly, we notice that the area where intraplate extension took place and the arc segment experienced a lull or reduction in magmatic activity^27^ coincides in space and time with the slab gap observed in the P-wave seismic tomography model (Figs. 1b,c and 3a).

To test the influence of the development of a slab gap in the magmatism of the Neuquén basin, we compare geochemical data from synextensional volcanic rocks of the Pre-Cuyano deposits^49–51,55^ with Cenozoic magmatism from the Tyrrhenian Sea and Apennine belt, which is one of the best verified examples of active slab tear settings. Rosenbaum *et al*. used the LILE/HFSE elements ratio (e.g., La/Nb) to analyze mantle metasomatism in different geodynamic settings along the Tyrrhenian Sea and Apennine belt. Variable La/Nb values were found in mixed slab tear-arc domains and slab break-off geodynamic settings. In contrast, low values were associated with asthenospheric upwelling through vertical slab tears producing a lesser degree of subduction-related mantle metasomatism in the arc^2^.

Compiled geochemical data from the Pre-Cuyano synrift show La/Nb ratios (average = 3.18) mainly ranging from 1.05 to 4.98. The samples plot across different possible geodynamic setting (see methods section for details) (Fig. 3b). We observe that most of the samples tend to concentrate on the lower range of La/Nb values coincident with the slab tear field (Fig. 3b). Specific geochemical relationships^57^ for discerning a slab break-off environment (La/Yb vs. Sr/Y; Nb/Y vs. Sr/Y; Nb vs. Y; Ta vs. Yb) do not indicate a dominant role for this process in the Pre-Cuyano synextensional magmatism (Fig. S3 in supplementary material). The results are compatible with the development of a slab rupture in Late Triassic-Early Jurassic times. This process and the resulting slab gap would explain the asthenospheric upwelling with arc influence suggested in previous studies^49,50^.

**Figure 4:**
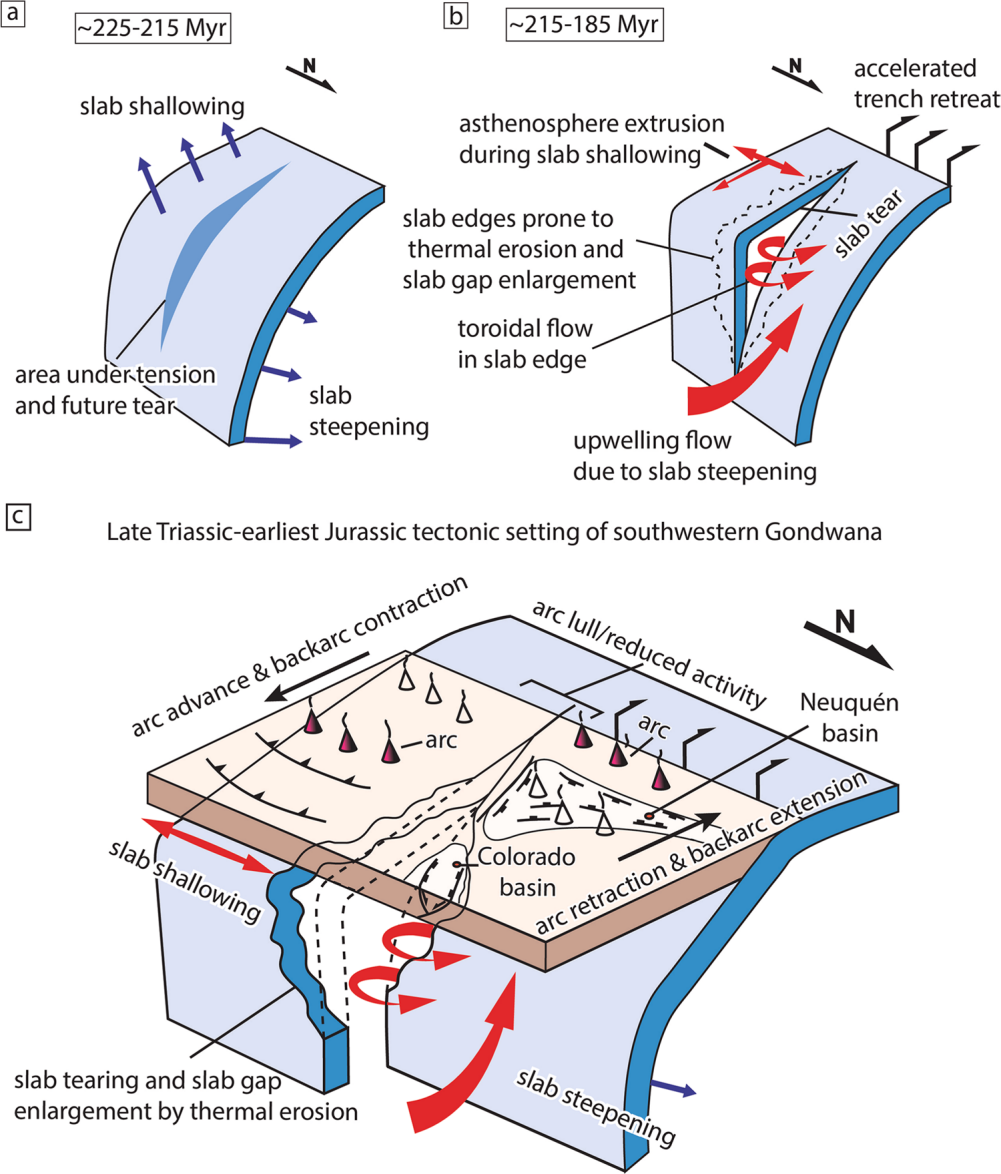
A conceptual model for slab-tearing beneath the southwestern Gondwana margin. (**a**) Slab-tearing in Late Triassic-earliest Jurassic resulted from synchronous development of slab shallowing in the south and slab steepening in the north inducing tensional stresses in a narrow zone ultimately leading to a slab gap. (**b**) Expected geodynamic response to the slab-tearing event and expected mantle flow patterns. (**c**) Late Triassic-Early Jurassic tectonic setting of southwestern Gondwana showing the contrasting tectonomagmatic evolution to the north and south of the inferred slab-tear.

Based on model predictions of sub-vertical slab gaps and observations in current settings, we suggest that creation of this discontinuity could have directly influenced the dynamics of the southwestern Gondwana margin. This process would have triggered mantle flow as a combination of toroidal flow through the slab gap and upwelling induced by the contemporaneous slab steepening north of 41°S, ultimately leading to accelerated rollback, a localized arc disruption and intraplate extension in the Neuquén and Colorado basins (Fig. 4).

A possible mechanism for the development of this slab gap is the interaction of a spreading ridge with the active margin. Unambiguous elements to identify this process are forearc anatexis and MORB magmatism at the site of ridge-trench interaction known as the ‘*blowtorch effect’*^58^ due to direct mantle upwelling beneath this area and the emplacement of forearc ophiolites indicating forced subduction of buoyant and young oceanic floor^23^. In the case where mid-ocean ridges interact obliquely with the margin, ridge-trench triple junctions migrate along the subduction zone and the time-transgressive tectonomagmatic effects are a hallmark signature of spreading-ridge subduction^23,46^.

As slab-tear events and ridge subduction share similar tomographic signatures and geological effects associated with arc shut-off and anomalous magmatism in the backarc area^23^, the lack of the key forearc tectonomagmatic record in the study area makes discriminating between both processes challenging^2,10,13,20–22^. Hence, establishing or discarding the scenario of ridge-trench interaction requires further studies documenting MORB and felsic magmatism and ophiolites in the forearc. More importantly, the stationary character of the tectonomagmatic evolution in the study area associated with a localized arc lull and synextensional activity lasting ~40 Myr contrasts the common time-transgressive nature of ridge-trench interactions^46^. A possible scenario compatible with the geological record would be that of a long spreading centre subducting at high-angle to the trench in a similar location for up to 40 Myr. However, recent numerical modeling of ridge subduction in a global 3D spherical setting indicates that ridges interacting at high angles with the trench (>60°) do not tend to develop slab window opening^59^.

Another potential mechanism is the interaction with an oceanic fracture. However, recognizing the past existence of these features is difficult, unless a remnant of the fracture zone is still preserved onboard the oceanic plate, which is not the case here. Besides, oceanic fractures do not always result directly in a slab rupture; instead, these mostly act as plate weaknesses that are eventually exploited by processes that induce slab rupture^16^. An alternative explanation is the subduction of an aseismic ridge^5^ or oceanic plateau^60^; however, there is no geological evidence of accreted seamounts that would support this past interaction^61^.

Alternatively, we suggest that the origin of the slab gap can be explained within the general geodynamic evolution of the southwestern Gondwana margin. The spatiotemporal analysis reveals a dramatic reconfiguration of the subduction system between ~225 and 190 Myr. At this time, north of the 41°S latitude there was a retraction of the magmatic arc, whereas to the south the arc system followed the opposite evolution (Fig. 2b,c). In the northern segment, arc retraction was likely associated with a slab steepening process produced by destabilization of a previous shallow slab configuration^40,62^ (Fig. 2b). In the southern segment, the arc migration was interpreted as related to the development the South Gondwanan flat-slab event, a large-scale flat-subduction episode that caused widespread intraplate contraction simultaneous to arc expansion^30^ (Fig. 2c). Such dramatic reconfiguration implies a lithospheric stretching of the area in between the slab segments rotating in opposite directions and the further development of a slab rupture that allowed for continued rotational plate motion (Fig. 4a,b). Noteworthy, mantle upwelling due to slab steepening is compatible with the development of a lithospheric dome linked to the formation of the regional Huárpica erosion surface^49^ that preceded the onset of extension in the Neuquén basin. We suggest that inherited structures in the upper-plate linked to a Paleozoic terrane suture^63^ could have also aided in the slab rupture (Fig. 3a). It is conceivable that in the context of the Late Triassic subduction reconfiguration a reactivation of this structure could have induced stress heterogeneities in the slab contributing to the rupture of the subducting plate.

We speculate that this major subduction reconfiguration is indirectly related to the formation of the Late Triassic South Gondwanan flat-slab. Seismic anisotropy observations in current flat-subduction settings have suggested that lateral and frontal squeezing of the asthenosphere takes place above the low-angle slab^64^. Similarly, we consider that the South Gondwanan flat-slab would have generated a trench-parallel supra-slab asthenospheric flow component that favored, and probably induced slab steepening of a pre-existing flat-slab^40,62^ north of the 41–42°S (Fig. 4).

In summary, our data support the hypothesis that a Late Triassic-Early Jurassic slab tear event in southwestern Gondwana caused a lower mantle slab gap, a contrasting spatio-temporal magmatic evolution, a local lull in arc activity^27^, the E-W intraplate extensional system related to the Neuquén and Colorado basins^29,48^, and anomalous magmatic activity^49–51,55^ (Fig. 4c).

This study provides an example of a holistic approach that can be used to identify vertical slab-rupture events in the Mesozoic world. Finally, we stress that identifying similar past slab-tearing events is of major importance as these processes control subduction dynamics and the overall tectonic regime^65^, which in turn governs the distribution of natural resources formed in convergent settings^22,66^.

## Methods

### Geochronologic datasets used in the spatiotemporal analysis

The position from the trench of the Carboniferous-Permian to Middle Jurassic magmatic arc in southwestern Gondwana as plotted in Fig. 2, is based on three previous compilations from Navarrete *et al*.^30^, del Rey *et al*. and Vásquez *et al*.^41^. These datasets are based on compilations of available radiometric ages (U/Pb, Ar/Ar, K/Ar, Rb/Sr) from Carboniferous to Jurassic plutonic and volcanic rocks with a subduction-related geochemical signature along southern South America and the Antarctic Peninsula. For clarity in our time-space diagrams, we plotted magmatic stages immediately before the arc shifting stages. Hence, arc position north of 41°S starts in the Carboniferous, while in the south it starts in the Permian. Geochronologic data and arc distances used in the space-time diagrams in Fig. 2b–d, are provided in the supplementary date (Table S1). Distance to arc rock ages was plotted perpendicular to the reconstructed margin. We did not take into account shortening in the upper-plate and subduction erosion. Hence, plotted arc-to trench distances represent a minimum value.

### Construction of geochemical fields

We extracted La/Nb ratios from Rosenbaum *et al*. for the different Italian magmatic environments linked to slab-tear, mixed slab-tear with subduction, slab break-off and undisturbed arc. In Fig. 3b, we plotted the approximate La/Nb ratios for each tectonic setting; slab-tearing (~1–4.5); continental subduction arc (~1.7–8); mixed subduction arc and slab-tearing (~1.8–7), and slab break-off (~2.5–9). Rosenbaum *et al*. do not explain why slab break-off and slab tear yield different La/Nb ratios. In this regard, numerical modeling and geochemical studies indicate that these two processes result in contrasting dynamics in the subduction system, mantle flow pattern, and related mixing of the mantle. In slab break-off settings, dehydration and melting of the tip of the detaching slab is more likely the main cause for magmatism^57^, which may explain the more variable La/Nb ratio^2^ in this setting. On the other hand, in slab-tearing settings the amount of mantle material exchanged between the arc and sub-slab mantle is more significant than in slab break-off events^66,67^, which probably explains the more marked asthenospheric signature in the latter setting^2^.

In our plot, we differentiate samples with basic to intermediate composition to provide a closer idea of the magma source, as isotopic data from the Pre-Cuyano synrift units is limited. Geochemistry data used in the Nb vs. La/Nb diagram in Fig. 3b is provided in the supplementary data (Table S2).

## Supplementary information

Tomotectonic and geochemical analyses

Dataset 2

Dataset 1
