# Supplementary material for: Surface and mantle records reveal an ancient slab tear beneath Gondwana
Source: Sci Rep. 2019 Dec 24;9:19774. doi: 10.1038/s41598-019-56335-9 (PMC6930287; doi:10.1038/s41598-019-56335-9)
Supplement: Supplementary file 1 — Tomotectonic and geochemical analyses [file 41598_2019_56335_MOESM1_ESM.pdf]

Scientific Reports

Supporting information for

Surface and mantle records reveal an ancient slab tear beneath Gondwana

Guido M. Gianni[1,2,3]*, César Navarrete[3], Silvana Spagnotto[4]

[1] *Instituto de Estudios Andinos Don Pablo Groeber, UBA-CONICET, Departamento de Ciencias Geológicas, FCEN, Laboratorio de Geodinámica. Universidad de Buenos Aires, Argentina. email:* guidogianni22@gmail.com

[2] *Instituto Geofísico Sismológico Ingeniero Volponi, Universidad Nacional de San Juan, Ruta 12, Km 17, CP 5407, San Juan, Argentina.*

[3] *Laboratorio Patagónico de Petro-Tectónica. Universidad Nacional de la Patagonia "San Juan Bosco", Dpto. de Geología, F.C.N., Argentina*

[4] *FCFMyN, Universidad Nacional de San Luis--CONICET, San Luis, Argentina*

Content of this file

Figure S1
Figure S2
Figure S3

Lower mantle vote maps

The consistency of the mantle structure and the slab gap analyzed in this work is supported by a high-velocity vote map presented in Fig. S1. We built this map at a depth of 1500 km, 1800 km, 2100 km and 2700 km corresponding to depth associated with Mesozoic subduction zones beneath southwestern Gondwana[37]. We generated this figure from stacking 24 global seismic tomography models; nine P-wave tomography models (Hosseini2016; GyPSuM-P; PRI-P05; SPani-P; GAP-P4; LLNL_G3Dv3; MITP08; MIT_USA_216MAY; UU-P07) and fifteen S-wave tomography models (GyPSuM-S; PRI-S05; SPani-S; S362ANI+M; S20RTS; S40RTS; SAVANI; SAW642ANb; SEMUCB-WM1; SEMum; SGLOBE-rani; TX2011; TX2015; SEISGLOB1; SEISGLOB2) using the web-based tool of Hosseini et al.[67] implementing a zero threshold metric. This methodology allowed us to identify where the models agree based on an increasing vote count[36]. Figure S1 confirms the presence of high anomaly velocities as indicated by high vote counts that were previously interpreted as fossil slabs[37] and a discontinuity interpreted as a slab gap shown at 2100 km represented by an area with low vote counts.

Reconstructions considering additional slab sinking rates

In Fig. S2 we provide alternative plate tectonic reconstructions taking into account faster slab sinking rates. For this, we use as a reference the studies of van der Meer et al.[24,37] that obtained a first-order estimate of the average slab sinking rate of 12 ± 3 mm/yr (see Fig. 1 in van der Meer et al.[24]), a range compatible with later empirical estimates (13 ± 3 mm/yr[38]; 9-12 ± 2 mm/yr[26]). We first carry out a fast sinking reconstruction considering the upper range value error of the average slab sinking rate (Fig. 2Sa-c) and another reconstruction considering the average value (Fig. 2Sd-e). In the former model, the slab gap below the study area is present up to Late Jurassic times and subduction of the South Orkney slab starts in the Early Cretaceous (~130 Ma), which is not consistent the geological record of active subduction before that time[44] and cessation of rifting and beginning of thermal subsidence in the Neuquén basin between 180-175 to 140 Myr[27,49]. A better fit with these geological constraints is obtained when considering the average slab sinking rate of 1.2 cm/yr (Fig. S2d-e), which indicates the existence of the slab gap below the Neuquén and Colorado basins in Late Triassic times and the subduction of the south Orkney Island slab at ~150 Ma that is partially compatible with the geological record indicating subduction activation between 180-170 Myr[44,45]. The best results are obtained with a slower sinking model considering a lower range value of 1 cm/yr presented in Fig. 1b-d. Hence, slab sinking rates between 1,2 and 1 cm/yr best relate to the geological constraints of southwestern Gondwana, which is consistent with studies analyzing subduction of ancient slabs around the world with global seismic tomography[24,26,36-39].

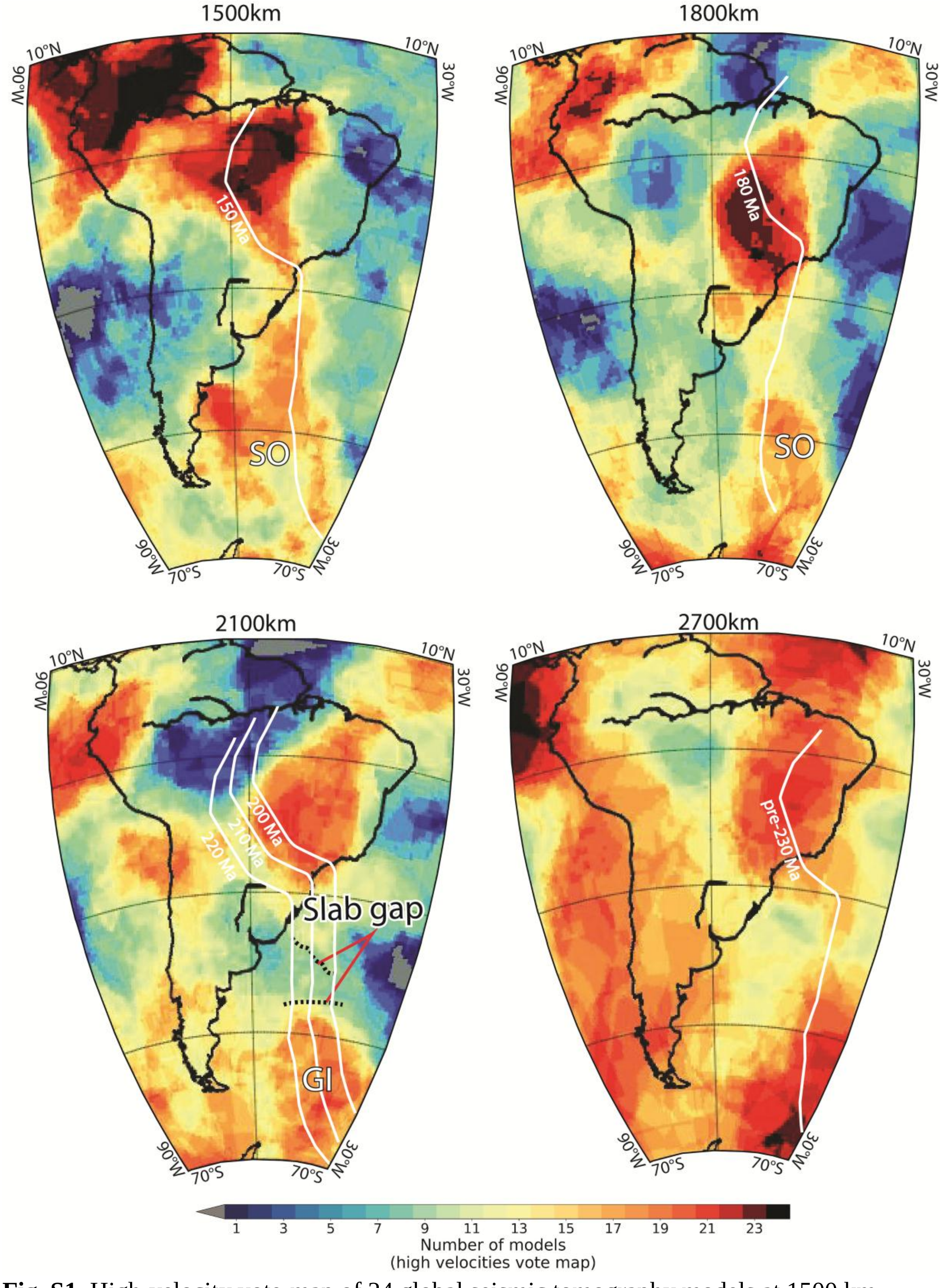

Fig. S1. High-velocity vote-map of 24 global seismic tomography models at 1500 km, 1800 km, 2100 km and 2700 km showing the consistency of the lower mantle structure beneath the study area. This figure shows the presence of a lower mantle high velocity

anomaly related to the Georgia Islands (GI)[24,25] and the slab gap discussed in this study. Also, we show positions of the southwestern Gondwana trench overlapped on these maps considering slab sinking rates of ~1 cm/yr[37] used in this study.

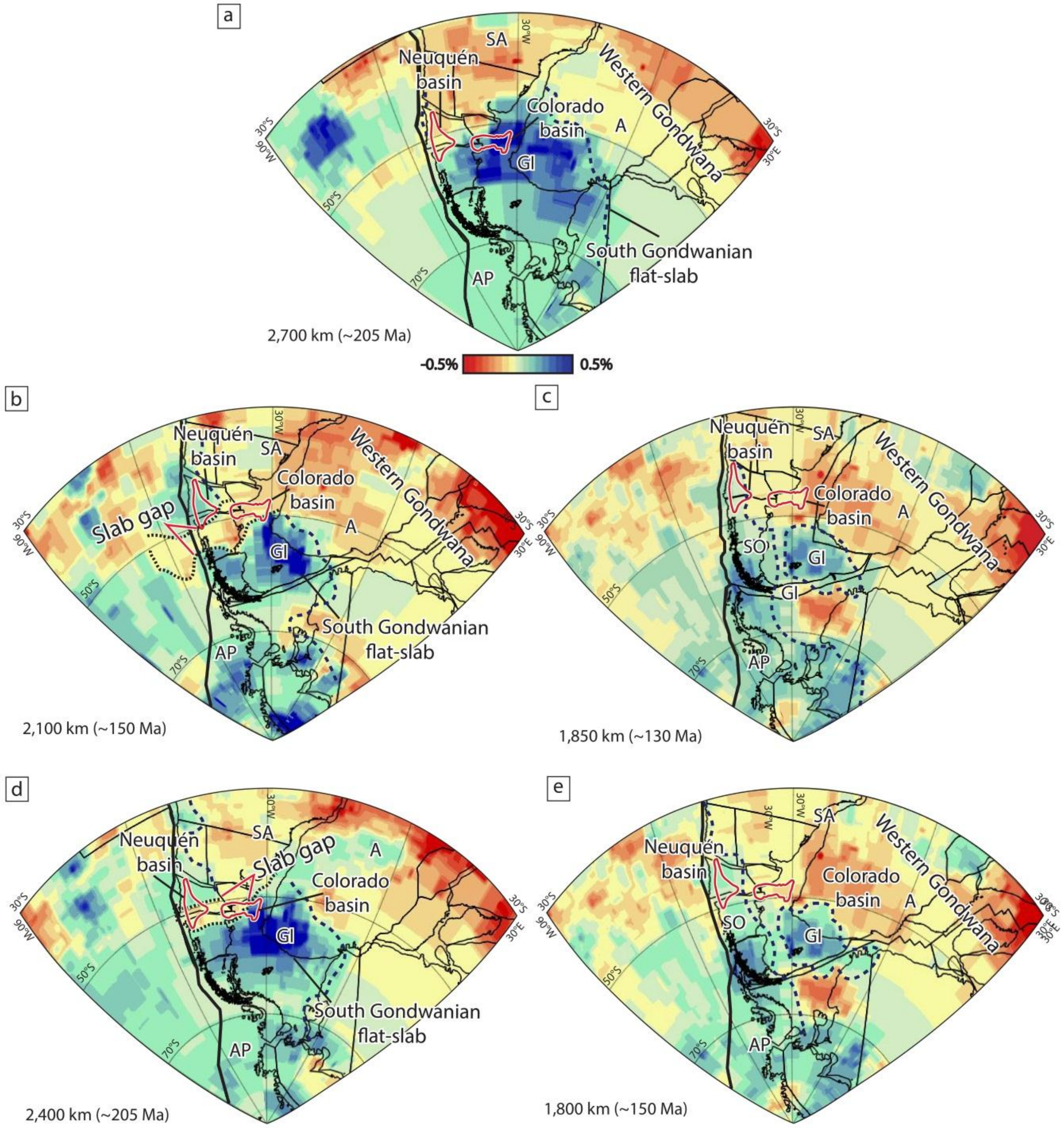

Fig. S2. Plate kinematic reconstructions and mantle analysis taking into account additional slab sinking rates. a-c) Fast sinking reconstruction considering the upper range value error of the average slab sinking rate (see Fig. 1 in van der Meer et al.[24]). d-e) Reconstruction considering the average slab sinking rate (12 ± 3 mm/yr)[24,37].

Additional geochemical analysis

To differentiate a slab break-off signature in samples from the Pre-Cuyano synrift unit we used the discrimination diagrams of Hildebrand et al.[57]. These authors suggested a series of geochemical relationships that allow discriminating an arc environment (or a magmatism that inherits the geochemical arc signature) from a slab break-off environment (e.g., La / Yb vs. Sr / Y; Nb / Y vs. Sr / Y; Nb vs. Y; Ta vs. Yb). We have used these diagrams for our compiled geochemical data and there is a clear predominance of geochemical relationships that rule out a slab break-off environment for the Pre-Cuyano synrift unit (Fig. S3). Hence, we suggest that the low La/Nb ratios[2] are compatible with an upwelling mantle produced by a vertical slab rupture (slab tear) (Fig 3b), which induces the partial melting of an asthenospheric mantle, with a variable arc influence most likely inherited from previous subduction events[55].

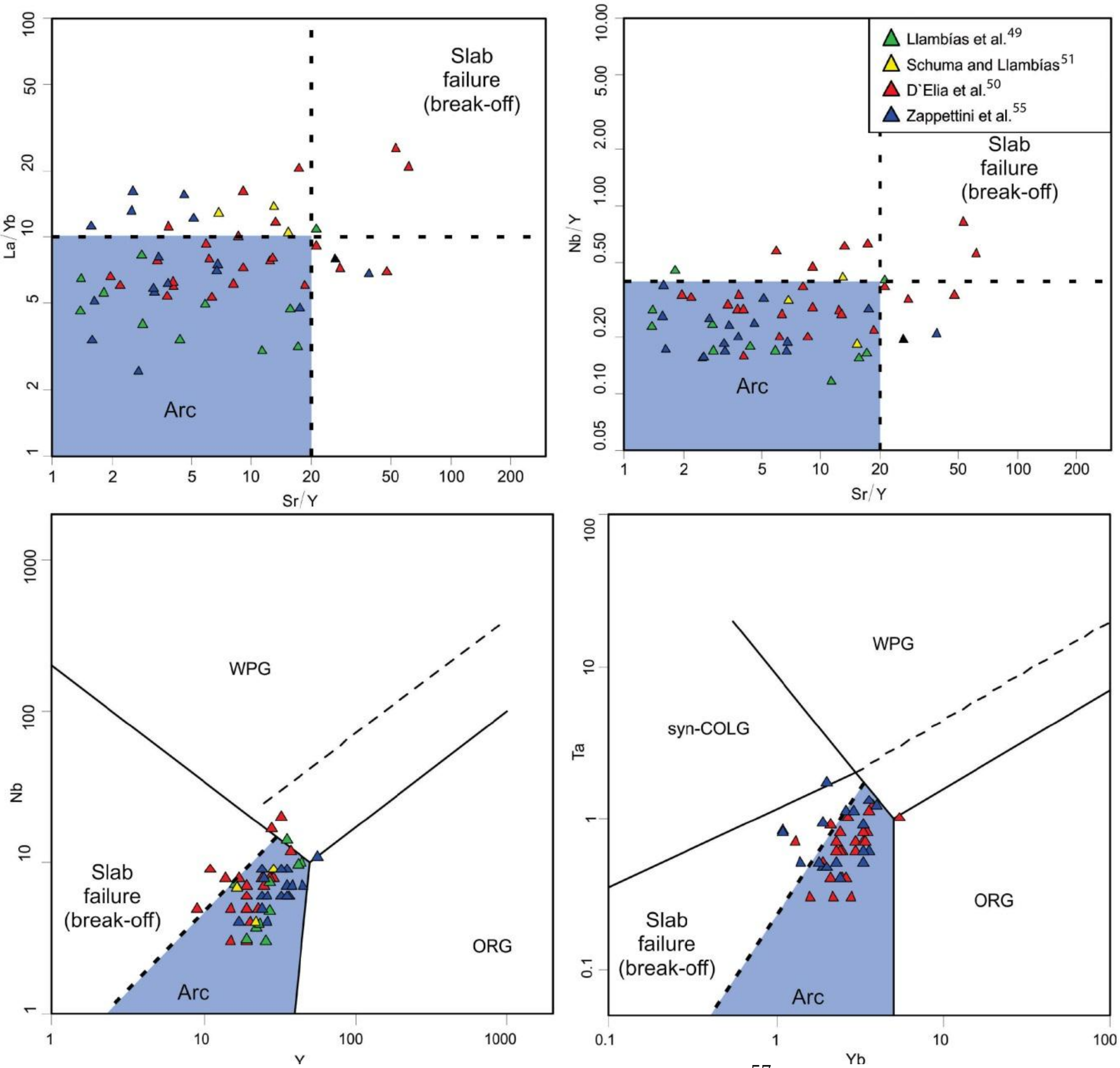

Fig. S3. Geochemical diagrams proposed by Hildebrand et al.[57] to discriminate magmatic arc and slab break-off environments in samples from the Pre-Cuyano synrift in the Neuquén Basin. The compiled geochemical data shows a magmatic arc geochemical

signature previously interpreted as a metasomatic inheritance in intraplate magmatism from the Permian subduction event[55].

References

67. Hosseini, K. et al. SubMachine: Web-Based tools for exploring seismic tomography and other models of Earth's deep interior. Geochem. Geophy. Geosy. 19 (2018).
